# Knowledge Versus Understanding: What Drives Moral Progress?

**DOI:** 10.1007/s10677-024-10465-w

**Published:** 2024-09-17

**Authors:** Petar Bodlović, Karolina Kudlek

**Affiliations:** 1 Institute of Philosophy, Ulica grada Vukovara 54, Zagreb, EU Croatia; 2 Ethics Institute, Department of Philosophy and Religious Studies, Utrecht University, Utrecht, The Netherlands

**Keywords:** Moral progress, Moral knowledge, Moral understanding, Evolutionary morality, Trolley problem, Doxastic change

## Abstract

Moral progress is often modeled as an increase in moral knowledge and understanding, with achievements in moral reasoning seen as key drivers of progressive moral change. Contemporary discussion recognizes two (rival) accounts: knowledge-based and understanding-based theories of moral progress, with the latter recently contended as superior (Severini 2021). In this article, we challenge the alleged superiority of understanding-based accounts by conducting a comparative analysis of the theoretical advantages and disadvantages of both approaches. We assess them based on their potential to meet the following criteria: (i) moral progress must be possible despite evolutionary and epistemic constraints on moral reasoning; (ii) it should be epistemically achievable to ordinary moral agents; and (iii) it should be explainable via doxastic change. Our analysis suggests that both accounts are roughly equally plausible, but knowledge-based accounts are slightly less demanding and more effective at explaining doxastic change. Therefore, contrary to the prevailing view, we find knowledge-based accounts of moral progress more promising.

## Introduction

The belief that individual and societal moral progress is possible is central to our understanding of morality and underpins many ubiquitous social practices. Parents instill moral values in their children with the hope that they will grow into better people. Legislators enact new laws that more accurately reflect moral values, assuming that adherence to these laws can morally improve society. Ethicists work on existing and new moral theories assuming that, in principle, moral theory can evolve and positively influence current moral practices. Without the assumption that moral progress is both possible and desirable, moral education, inquiry, social learning, and legislative reform would lose much of their purpose.

While the concept of moral progress faces significant challenges (see Buchanan and Powell 2018, pp. 1–4), some examples of such progress are indisputable, necessitating a systematic approach to understand them. For instance, slavery was once widely accepted, but it is now almost universally condemned. Women were denied the right to vote, but now most countries recognize their equal rights. A theory of moral progress is crucial for explaining how positive moral change comes about, and how moral norms can be sustained and promoted. Not only do plausible accounts of moral progress explain historical instances of moral change, but also provide practical, normative guidance for present and future moral development, including policymaking and social reform.

In this paper, we focus on individual moral progress. Contrary to supra-individual, societal or group-level progress, individual moral progress is best understood as an improvement or development in the moral capacities of individual agents. This development is usually described as a positive, durable, non-superficial, and irreversible change in various components of moral functioning^1^ such as the improvement of moral reasoning (Schinkel and de Ruyter 2017). More broadly, the improvements can happen in epistemic, decisional, and motivational capacities (Bina et al. MS). When it comes to improvements in epistemic capacities, moral progress consists of agents getting better at moral reasoning, grasping moral concepts, or beliefs getting better (Moody-Adams 1999; Songhorian et al. 2022; Bina et al. MS). What especially matters for our discussion here is *how* agents improve their moral beliefs—be it abandoning old, acquiring new, or revising existing beliefs.

Also, we focus on moral reasoning as one of the cornerstones of morality (see, e.g., Buchanan and Powell 2018, p. 59) and the most discussed drivers of moral progress. Moral reasoning is often thought to be a good explanation for at least some of the moral progress that has occurred throughout human history (Singer 2011; Campbell and Kumar 2012).^2^ Some scholars even believe that it is a preferable way of achieving moral progress because moral attitudes acquired via a rational approach are more reliable and durable (Kumar and May, Forthcoming). Moral reasoning has progressive potential because it allows people to identify and resolve moral inconsistencies (Killen and Dahl 2021).

But moral reasoners can be oriented towards different goals. For instance, *moral knowledge* and *moral understanding* represent distinct goals, functions, or outcomes of moral reasoning and both play a significant role in explaining how individual moral progress comes about, especially when embedded with other factors such as social and structural changes (e.g., Kumar and May, Forthcoming).^3^ Accordingly, the paper’s central question is: to achieve (epistemic) moral progress, should the *individual reasoner* seek *understanding* rather than *knowledge*? Is (epistemic) moral progress better explained through developing intellectual abilities to solve new problems and moral dilemmas (i.e., understanding), or through the accumulation and transmission of warranted true beliefs (i.e., knowledge)? More technically: are understanding-based accounts of moral progress (henceforth, UAP) theoretically superior to knowledge-based accounts of moral progress (henceforth, KAP), or vice versa?

We acknowledge the potential of both knowledge-based and understanding-based theories, but argue that KAP are slightly more promising. While much of our discussion questions the alleged superiority of UAP advocated by Severini (2021), our ultimate goal is constructive: enhancing the debate by examining how understanding and knowledge relate to and facilitate moral progress. The paper highlights several issues overlooked by Severini (2021) that are, nevertheless, essential for developing a comprehensive epistemic account of moral progress. Consequently, our analysis is not merely a critique but offers valuable insights, even for those unfamiliar with Severini’s views.

The paper’s contribution is twofold. First, it opens much-needed new perspectives and research avenues that are yet insufficiently explored in the moral progress debate. Second, the concepts and arguments developed in this paper have direct implications for several ongoing debates.

As for the first part, the paper introduces three new challenges for UAP—evolutionary constraints, cognitive demandingness, and the difficulty of explaining a doxastic change—and shows that while KAP and UAP are, roughly, equally demanding when it comes to allowing moral progress, the former are slightly better at explaining the doxastic change. Furthermore, it analyzes the importance of knowledge and understanding from an epistemic and pragmatic, rather than ontological (metaethical) standpoint. Instead of discussing skepticism and the possibility of moral knowledge (see Severini 2021; Sauer et al. 2021), the paper provides a normative comparison of knowledge and understanding, aligning with the non-ideal turn in philosophy that, roughly, prefers to study non-ideal agents under suboptimal circumstances (see Valentini 2012). This also makes our analysis more relevant to practical philosophy readership.

Moreover, the paper highlights that there is more to moral reasoning and moral progress than merely acquiring true beliefs. True beliefs are necessary for knowing and relevant for understanding, but our approach emphasizes the importance of justifying one’s beliefs, being sensitive to new evidence, discriminating between relevant and irrelevant premises, inferring correct conclusions in new epistemic circumstances, or reasonably retracting, revising, or retaining moral attitudes. These elements are crucial for a comprehensive, epistemologically informed account of moral progress. Finally, our paper shows that moral progress *via* understanding requires consistently applying moral principles to *new situations*, even when such situations do not include “expanding the moral circle” (Sauer et al. 2021) to people of different sex, gender, race, ethnicity, sexual orientations, etc., or non-human animals (see Sect. 5.1).

As for the second part, a notion of moral understanding developed in this paper might imply that some types of moral progress should be studied individually, rather than socially (see Sect. 5.2); expanding the moral circle may rest on the agent’s ability to morally understand (see Sect. 5.1); and understanding may be more essential to moral motivation than knowledge, thereby contributing to another basic type of moral progress (see Sect. 5.2).

Questions concerning human cognitive limitations, consistency preservation, doxastic change, or reasoning in new circumstances (indirectly) underlie many ethical discussions and are answered differently depending on whether a moral reasoner seeks knowledge or understanding. The same holds for the question of whether reasoning should produce a stable cognitive achievement, or some epistemic state that can be easily undermined by new evidence. Or should it produce relatively superficial, but actionable knowledge easily transferable to agents when action is urgent, or, by contrast, be associated with the ability to reflect deeply on eternal moral issues? The significance of previous questions, as well as the relationship between knowing and understanding, therefore, obviously goes beyond a very specific discussion concerning moral progress.

The paper proceeds as follows. We first outline features specific to each concept (Sects. 2 and 3), followed by aspects that set these two accounts apart (Sect. 4). Through identifying some main points of divergence between knowledge and understanding we proceed to challenge the alleged superiority of UAP by conducting a comparative analysis of both approaches (Sect. 5). We identify several theoretical advantages and disadvantages for both accounts in terms of their potential to satisfy the following criteria: (i) moral progress should be possible despite evolutionary and epistemic constraints on moral reasoning; (ii) moral progress should be epistemically achievable to ordinary moral agents without being overly demanding; (iii) moral progress should be explainable via the doxastic change.

## Moral Knowledge

Moral epistemologists are generally more interested in exploring the possibility of moral knowledge and its epistemic sources than in providing a detailed conceptual analysis (see Campbell 2019).^4^ They usually accept the traditional definition of knowledge as justified true belief.^5^ Consequently, “one cannot know that stealing is immoral unless it is true that stealing is immoral, one believes that stealing is immoral, and one’s belief is justified” (Sinnott-Armstrong 1996, pp. 5–6). This traditional framework is “widely accepted, including by many philosophers who differ significantly in their substantive accounts of knowledge and justification” (McGrath 2019, p. 7). Subtle differences aside, two distinctive features of knowledge are essential to our present analysis: defeater-sensitivity, and luck-sensitivity (see Hills 2016).

First, knowledge is sensitive to defeaters. Suppose that “Stealing is immoral” is true, John believes it, and possesses the following justification: “Stealing is immoral because Theresa—a competent and benevolent moral expert whose judgment is, generally, reliable—says that it is immoral.” In this case, John knows that “Stealing is immoral” until some counter-consideration undermines his inference. He might learn that stealing is morally desirable in “Robin Hood” situations, or that Theresa’s judgment about stealing is compromised (e.g., because, recently, her child was hurt during the robbery), or that equally competent moral expert, Mary, believes stealing is moral. Without eliminating such defeaters, John does not know that stealing is immoral: his true belief is unjustified.^6^

Second, knowledge is sensitive to epistemic luck. Imagine that John asks a random passer-by, Theresa, “Is stealing moral?” She assures him that it is not, and he defers to her testimony. But suppose, first, that John lives in a decadent society where deferring to Theresa’s judgment was extremely lucky: if asked the same question, almost any other passer-by would assert “Stealing is moral” and John would acquire false belief. Or, second, imagine Theresa understands the moral implications of stealing, but is an unreliable judge of other moral matters. Due to John’s general interest in morality, he could have easily asked her about lying, justice, or animal rights and would have acquired false beliefs. In both cases, epistemic luck seems relevant to ascribing knowledge. Since John’s true belief was acquired accidentally, by an *unsafe* process of belief-formation (Pritchard 2007), it cannot represent knowledge.

Finally, for our purposes, it is important to acknowledge a distinction between “knowledge that” and “knowledge why” (Hills 2009, 2016). John may know *that* “Stealing is immoral,” but can he really know *why* “Stealing is immoral?” based solely on Theresa’s reliable testimony? It doesn’t seem so. Although Theresa’s testimony justifies John’s belief about stealing, it does not, per se, *make* stealing immoral. What makes stealing immoral and provides genuine knowledge why, is, presumably, some further moral consideration.^7^ For instance, John may condemn stealing because it violates the right to private property, or creates violence and social insecurity. So, to generate knowledge why *p*, *q* must be somehow responsible (conceptually, causally, normatively) for *p* being true. The truth of *p* must directly depend on *q*.

## Moral Understanding

Understanding can be of different types and include different objects (Grimm 2021). One might understand how the computer works, how to bake a cake, what a concept means, that proposition is true (“I understand that you care”), and why proposition is true (“I understand why stealing is immoral”) (Hills 2016). Furthermore, one can understand people (“I understand John”), events (e.g., 9/11 attack), subject matters (e.g., chemistry), theories (e.g., theory of gravitation), single propositions (“Stealing is immoral”), and concepts (e.g., justice) (Elgin 2007; Hills 2016; Grimm 2021; Moody-Adams 1999). Following Hills (2009, 2016), our analysis focuses on understanding why a singular proposition is true, or, more precisely, grasping inferences such as “*p* because *q*.” Then, following Severini (2021), we explore the implications of “understanding why” and “knowledge why” for moral progress.

Understanding is a cognitive achievement or success (Hills 2009; Elgin 2007). While some scholars portray it as a cognitive relation (Elgin 2007) and others as an epistemic state (Grimm 2010; Hills 2016), “there seems to be widespread agreement that … understanding … essentially involves an element of ‘grasping’ or ‘seeing’” (Grimm 2010, p. 342). So, to understand why *p*, one must *grasp* the inference “*p* because *q*.” What does this intellectual ability, exactly, require?

Grasping “*p* because *q*” requires having this inference under “cognitive control” (Hills 2016), or appreciating it at the deeper intellectual level (Hills 2009, p. 101; Grimm 2021). Hills (2009, 2016) identifies several abilities that demonstrate cognitive control. First, there are *communicative abilities*, such as expressing a piece of inference in one’s own words. If John defers to Theresa’s inference “Stealing is immoral because it violates the right to private property,” he hardly demonstrates any intellectual control by merely quoting Theresa’s own words.^8^ Secondly, to understand “*p* because *q*,” one must be able to conclude *p* from *q* (if *q* seems true), and explain *p* in terms of *q* (if *p* seems true). Although these *direct inference abilities* seem straightforward, they concern a non-trivial ability to instantiate a piece of inference. For instance, to understand why stealing is immoral, John must be able to quote the violation of the right to private property when a thief steals his wallet, or a state nationalizes his land. So, generally, understanding is associated with “an adeptness in using the information” (Elgin 2007, p. 35) or “put[ting] the correct answer to use” (Grimm 2010, p 341). An ability to “apply the principle to particular situations” (Grimm 2010, p 341) demonstrates such adeptness.

Most importantly, grasping requires accurate reasoning in similar cases (Hills 2009, 2016). Such *analogical reasoning abilities* are best explained by an ability to manipulate variables (Hills 2016). As Grimm (2010, pp. 340–341) puts it, an agent should be able to “anticipate how changes in the value of one of the variables … would lead to (ceteris paribus) a change in the value of another variable.”^9^ Imagine Mark forgot to return John a considerable sum of money. Arguably, such behavior is similar but not identical to stealing, so does the original reason *q* (“John’s right to private property is violated”) still explain the modified conclusion p* (“Not returning borrowed money is immoral”), or should alternative explanations—such as broken promises, lack of responsibility, etc.—come to the fore? Next, imagine the original reason is slightly modified. If a private corporation makes public goods inaccessible to people entitled to them, presumably, the corporation’s action is immoral without violating the individual right to *private* property. Should, then, violation of some broader moral principle be preferred in explaining the immorality of stealing? In this sense, understanding “*p* because *q*” assumes an agent’s “capacity to answer new questions” (Scriven 1972, p. 32).

## Why is Understanding Different from Knowledge?

The relationship between knowledge and understanding is controversial. While reductionists argue that understanding is reducible to knowledge, non-reductionists treat them as connected, but independent epistemic achievements (Severini 2021, pp. 93–98). Since our present analysis rests on distinguishing knowledge from understanding, this section presents non-reductionist intuitions about the relevance of grasping (for knowledge), as well as truth, justification, defeaters, and luck (for understanding).

### Knowledge does not Require Grasping

Here’s the potential difference: understanding is primarily about one’s abilities, while knowledge isn’t. Traditional definitions of knowledge attribute justified true beliefs to epistemic subjects, but do not focus on their cognitive abilities.^10^ But just like understanding, knowledge must require some cognitive abilities and, if this is so, perhaps the whole proposition-centered analysis concerning justificatory relations might, in principle, be translated into an agent-centered analysis concerning justificatory abilities.

This brings us to the question: What kinds of cognitive abilities does knowledge require, i.e., does knowing why *p* require grasping why *p*? Prima facie, grasping why doesn’t seem necessary for knowing why. Namely, suppose that, at *t*_*1*_, Mark knows why plagiarizing John’s idea is immoral (namely, because plagiarizing violates one’s right to private property). However, at *t*_*2*_, Mark fails to competently evaluate a corporation’s usurpation of public property: variables change, so he draws wrong conclusions. This shows that Mark does not really grasp the original inference, but how can the wrong judgment regarding the corporation that steals public material property defeat Mark’s knowledge about the plagiarist who steals John’s private intellectual property? It seems that his knowledge cannot be threatened, so, presumably, the first essential difference between knowing and understanding is the following: knowing “*p* because *q*” requires some cognitive ability, but, technically, does not require grasping.

### Understanding does not Require Truth

While the first difference concerns the nature of cognitive abilities, the second concerns the truth values of relevant propositions. Factive accounts propose that, just like knowledge, understanding describes facts and, therefore, requires true beliefs (Grimm 2010; Hills 2016). By contrast, non-factive accounts allow that false propositions can also contribute to understanding (Elgin 2007).

The extreme non-factive accounts allow us to understand false propositions based on other false propositions. Suppose an astrologist predicts that John will fall in love this February because he is a Scorpio. Since one’s love life doesn’t essentially depend on the date of birth, key elements of explanation are false. Also, unsurprisingly, John does not fall in love in February. However, the astrologist is still able to manipulate the variables: for instance, she can take into account the time of birth, zodiac sign, positions of celestial bodies, etc., and predict what John’s love life would be if he were a Leo. The ability to draw ‘what if’ inferences while preserving coherence (not with real-world facts, but broader astrological framework) represents “a genuine cognitive achievement” (Elgin 2007). Astrology is madness, but there is still some method to it, and astrologist grasps this method very well.

Such radical account of non-factive understanding is connected to the so-called conditional grasping, where I grasp “that A depends on B … only in the light of certain assumptions that I either fail to accept or that I take to be importantly incomplete” (Grimm 2010, p. 341). Despite their normative insufficiency, extreme non-factive accounts also enable us to explain some cases of genuine, or desired understanding. For instance, they agree with our intuitions that scientists *can* understand rejected theories (e.g., Phlogiston theory) and that, in some contexts, truthful explanations are simply not a priority. For instance, Nyrup and Robinson (2022, p. 13) mention the case of a terminally ill patient and suggest that, pragmatically, understanding that offers a meaningful and comforting narrative about the end of life should be preferred over understanding that reflects the harsh truth.

By contrast, moderate non-factive accounts acknowledge the positive connection between understanding and facts but interpret this connection as something other than (complete) correspondence. Elgin (2007) discusses two cases. First, *approximately true* propositions are legitimate objects of understanding. For instance, “Humans descended from apes” only approximates truth, but if one can explain it by applying biological studies of heredity and evolution, and coherently using concepts like natural selection, fitness, adaptations, common ancestors, etc., then one sufficiently understands human evolutionary origins (2007, pp. 37–41). Second, *fictive principles* can help us understand true factual statements. Some scientific idealization, such as an ideal gas law, might explain the actual behavior of some real gas. Science often generates understanding based on idealizations, although, strictly speaking, idealizations are false (2007, pp. 38–41). Along these lines, de Regt (2015, p. 3782) remarks that a physicist who relies on Newton’s theory of gravitation *understands* the interaction between particles, although Newton’s key hypothesis—the existence of attractive forces—is false, i.e., defeated by Einstein’s theory of general relativity.

To conclude, moderate non-factive accounts acknowledge that, in some sense, understanding answers to facts, but deny its “slavish sensitivity to the facts” (Elgin 2007, p. 33). This makes understanding different from knowledge.

### Understanding does not Require Justification

The third difference between knowing and understanding focuses on justificatory relations. Unlike knowing, understanding why is not sensitive to defeaters and epistemic luck: presumably, one can grasp “*p* because *q*” even if *q* does not justify *p*.

First, suppose the astrologist predicts John will fall in love based on astrological considerations. Since scientific evidence undermines astrology, the astrologist cannot *know* why John will fall in love (even if the prediction turns out to be true). As non-factive accounts suggest, however, she can still understand why John will fall in love in a (minimal) sense of being able to manipulate variables in an internally consistent fashion. So, although scientific evidence defeats astrological explanation, it does not defeat the understanding provided by it. As Lipton puts it, “a new competitor may decrease the likeliness of an old hypothesis, but it will usually not change its loveliness” (2009, p. 60).

Second, understanding is not sensitive to misleading evidence. Let us reformulate the example found in Hills (2016). Imagine John believes that stealing is immoral because it violates the right to private property but Theresa, a trustworthy moral expert, disagrees. John irresponsibly ignores Theresa’s opinion, but, surprisingly, her judgment turns out to be false and entirely irrational. While ignoring Theresa’s misleading testimony affects John’s justification, it does not affect his understanding. John cannot *know* why stealing is immoral because he irresponsibly ignored relevant evidence, but he can still understand why this is so: John is able to provide an explanation and grasp the connections between true propositions. So, both factive and non-factive accounts entail that understanding might be immune to epistemic defeaters.

Third, understanding does not seem sensitive to epistemic luck. Let us slightly reformulate Hills’ (2016) example. Imagine that during the lecture about stealing your ethics professor says: “That is immoral because it violates one’s right to private property…” While you naturally assume she refers to stealing, she is, in fact, commenting on a similar case where Mark forgets to return money to John. Since your true belief that “Stealing violates one’s right to private property” is acquired accidentally—as a result of “Gettier luck” and epistemically irresponsible behavior—you cannot, strictly speaking, *know* why stealing is immoral. However, you can still understand why this is so: your conclusion is true, supported by a true premise (that you can quote), and having “Gettier luck” does not prevent you (at least not in principle) from competently manipulating variables and grasping the connection between *p* and *q*.

In summary, knowledge and understanding are desirable but represent distinct epistemic achievements. While knowledge is propositional (although it requires abilities), understanding is a cognitive ability (that requires propositions). Unlike understanding, knowing does not require grasping and, unlike knowing, understanding does not strictly require truth and justification. This brings us to our main question: shall we model moral progress in terms of knowledge or understanding?

## Moral Progress between Knowing and Understanding

In this section, we discuss comparative theoretical (dis)advantages of knowledge and understanding for developing an account of moral progress. We identify three criteria that a plausible account should satisfy.

1. It must make moral progress *possible* in light of the evolutionary origins of morality (and our epistemic limitations).
2. It shouldn’t be overly *demanding*: moral progress should be epistemically achievable by ordinary agents.
3. It must be able to explain moral progress via *doxastic change.*

We use these criteria to assess UAP, thereby conducting a kind of theoretical analysis that, to our knowledge, has not been conducted before. However, this list is by no means exhaustive; a successful theory of moral progress must meet many requirements (see, e.g., Buchanan and Powell 2018, p. 31) and our conclusions are just part of the broader picture. The first criterion reflects the intuition that “a theory of moral progress ought to be compatible with the relevant psychological and social facts about human beings” (Buchanan and Powell 2018, p. 27). The second one further emphasizes the need for a non-ideal, i.e., realistic instead of utopian theory of moral progress (see Valentini 2012). Our third criterion is an original contribution to the discussion emphasizing that beyond explaining specific, paradigmatic cases like the reduction of slavery, a plausible theory of moral progress must also account for the general *cognitive mechanisms* underlying moral progress. This includes the processes of abandoning/revising old beliefs, or adopting new ones in response to new evidence. Importantly, we show that epistemic obstacles to belief revisions arise not only from the cognitive limitations of moral agents (which is the standard approach in the literature), but also from the very nature of epistemic goals (such as knowledge and understanding) that agents pursue.

We argue that KAP and UAP are equally plausible when it comes to allowing moral progress (Sect. 5.1), but that KAP are slightly less demanding in some respects (Sect. 5.2), and better at explaining doxastic change (Sect. 5.3).

### UAP are (not) Challenged by Evolutionary Origins of Morality

Let’s start by presenting a recent, three-step argument in favor of UAP offered by Severini (2021). The first step—the *Evolutionary Debunking Argument—*attempts to show that humans cannot have moral knowledge since natural selection does not aim at true beliefs.

[Moral beliefs] emerged through natural selection because this way of thinking provided our ancestors with some sort of survival and reproductive advantage. If so, the evolutionary explanation of morality undermines the epistemic standing of moral beliefs by showing that those beliefs were formed by an unreliable process, insofar as natural selection aims at fitness, not at detecting some mind-independent moral facts. Thus, once we recognize that evolutionary forces have shaped our moral beliefs, we become less confident that those beliefs are true; and we legitimately come to doubt that we can have moral knowledge. (Severini 2021, p. 91)

But if we cannot have any knowledge, then, obviously, we cannot accumulate moral knowledge and KAP make moral progress impossible. Since the evolutionary origins of morality “cannot be rejected” (Severini 2021, p. 92), we should develop some alternative account of moral progress.

Severini believes that defining moral progress in terms of increased understanding avoids the *Evolutionary Debunking Argument*: “The main theoretical gain of the understanding-based account of moral progress lies in its being perfectly consistent with the evolutionary explanation of morality” (Severini 2021, p. 98). What lies at the heart of her *Evolutionary Consistency Argument* (our label) is the non-factive nature of understanding. Since understanding neither requires nor necessarily produces true beliefs (see Sect. 4.2), fitness-directed features of moral reasoning cannot undermine it. The non-factive notion of understanding makes evolutionary considerations toothless, and moral progress possible.^11^ Based on the evolutionary debunking of knowledge and evolutionary consistency of understanding, in the third step of her argument, Severini concludes that UAP are comparatively superior. For clarificatory purposes, we present her argument schematically.


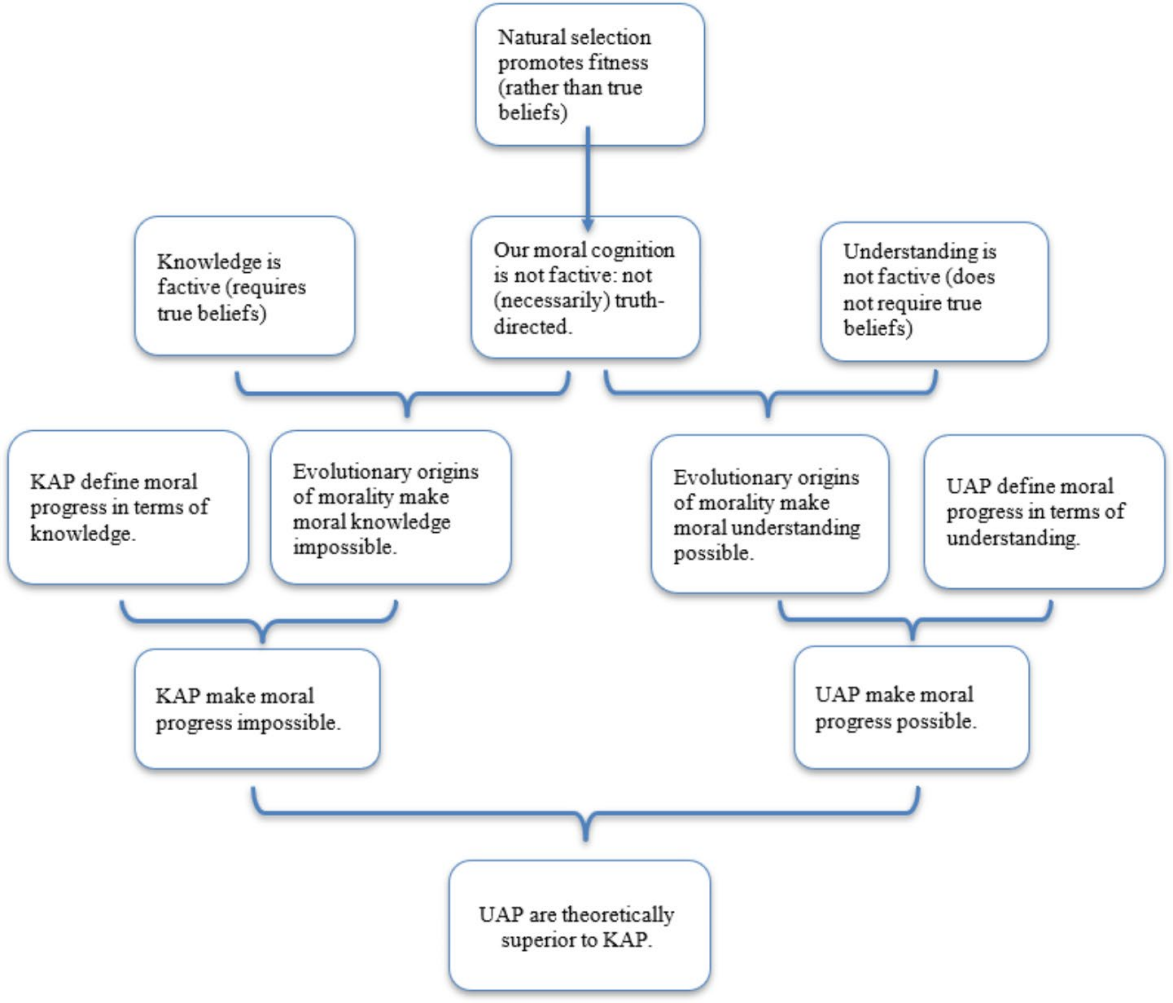


But do evolutionary explanations of morality make UAP superior to KAP? We don’t believe they do. Insofar as Severini’s *Evolutionary Debunking Argument* is concerned, the skeptical conclusion seems hasty because promoting fitness is typically consistent with and may even require a reliable cognition: the ability to acquire true beliefs via defeasible reasoning surely represents a reproductive advantage.^12^ The conclusion of the *Evolutionary Consistency Argument* seems hasty, too. That non-factive features of understanding are consistent with evolution does not mean that all other features are consistent, too. In other words, even if understanding does not require true beliefs, it surely requires *something*. So, are such requirements threatened by evolutionary explanations, or are understanding and evolution consistent through and through?

To tackle this question, let’s first distinguish psychological from epistemic requirements. Psychologically, understanding typically requires having the “Aha!” or “Eureka!” experience (Walton 2005; Grimm 2010) or a particular “sense” of understanding (Trout 2002). Epistemically, understanding why requires an ability to instantiate abstract principles, apply principles to new domains, and competently reason with changed variables (see Sect. 3). It is little wonder that our cognitive limitations (shaped by natural selection) pose challenges to “Aha!” experiences, as well as grasping abilities.

Psychologically, we often have a false sense of understanding. Trout (2002, pp. 223–229) explains this phenomenon by referring to overconfidence and hindsight,^13^ while Keil (2006) believes that overestimation comes from the “illusion of explanatory depth,” a specific cognitive bias that is not “merely another case of a general overconfidence effect” (Keil 2006, p. 13). But if cognitive biases related to overconfidence promote fitness (Johnson and Fowler 2011) while systematically undermining understanding, then the *Evolutionary Consistency Argument* seems rather weak. If evolutionary-shaped cognitive biases threaten both knowing and understanding, how can understanding, unlike knowing, remain consistent with natural selection?

Furthermore, notice that without the ability to discriminate between relevant and irrelevant variables, we won’t be able to competently reason in similar cases.^14^ However, extensive research in evolutionary and moral psychology suggests that moral judgments are affected by all sorts of morally irrelevant factors: from emotional responses (Greene et al. 2001), order and framing of experiments (Schwitzgebel and Cushman 2012), to irrelevant situational factors (Klenk and Sauer 2021). Such factors are obstacles to consistent reasoning, a key requirement of both moral progress (see Buchanan and Powell 2018, p. 55) and understanding (see Sect. 4.2). To illustrate how moral understanding can be challenged by evolutionary factors, we turn here to the data collected in solving sacrificial dilemmas, such as the well-known Trolley vs. Footbridge dilemma.

As Greene et al. (2001) show, people naturally apply the utilitarian maximization principle to the famous Trolley dilemma. Put simply, the utilitarian reasoning goes like this: if I must choose between hitting the switch to save five lives, or not hitting the switch to save one life, then I am morally obliged to hit the switch and save five lives. To truly understand why we should hit that switch, we should be able to produce correct judgments under changed circumstances, or variables. However, our abilities to apply abstract principles or reason consistently in similar cases seem challenged as soon as we face the so-called Footbridge dilemma. When, *ceteris paribus*, the original variable “hitting the switch” changes to “pushing someone off the bridge,” we usually hesitate to apply the utilitarian maximization principle: suddenly, killing one to save five is no longer morally acceptable. The inability to reason coherently across morally similar cases challenges our understanding of the original, utilitarian, inference.

Our moral understanding of sacrificial dilemmas is reduced by whatever stands in the way of coherent reasoning. But why, exactly, do we draw inconsistent conclusions in morally similar sacrificial dilemmas? The first explanation is related to different levels of emotional engagement.

We maintain that, from a psychological point of view, the crucial difference between the trolley dilemma and the footbridge dilemma lies in the latter’s tendency to engage people’s emotions in a way that the former does not. The thought of pushing someone to his death is, we propose, more emotionally salient than the thought of hitting a switch that will cause a trolley to produce similar consequences, and it is this emotional response that accounts for people’s tendency to treat these cases differently. (Greene et al. 2001, p. 2106)

Second, more recent research suggests that both controlled deliberation and automatic unconscious inference drive our resolutions of moral dilemmas (Kumar and May, Forthcoming; Greene 2014).^15^ Third, moral judgments about sacrificial dilemmas may also be influenced by the experiments’ order and framing (see e.g., Horne and Livengood 2017). If participants are first presented with the push-type dilemma, and conclude that pushing the person to death (to save five) is wrong, then they are also less likely to “hit the switch” (Schwitzgebel and Cushman 2012). Crucially, previous cognitive obstacles are easily explainable in naturalistic terms. Emotions have evolutionary origins and, in some respects, are advantageous (Tooby and Cosmides 2008) although they often cloud our judgment and trigger different types of motivated reasoning. Furthermore, automatic, unconscious reasoning is necessary from the cognitive economy standpoint and, thus, naturally selected despite being epistemically suboptimal (see Kahneman 2013). Finally, our unwarranted sensitivity to order and framing can also be explained in naturalistic terms, perhaps by referring to the so-called “anchoring bias.”

But do inconsistencies necessarily undermine understanding? Can’t inferring opposite conclusions somehow *indicate* understanding? For instance, moral agents must choose between two actions in sacrificial dilemmas both of which are, to some degree, justified. Since the decision is tough, doubts and inconsistencies are natural and might indicate, rather than undermine moral understanding. Although this observation seems plausible, it does not refute our conclusions and may even strengthen them. We offer four remarks for the sake of further clarification.^16^

First, we do not argue that inconsistencies necessarily *undermine* understanding. Such a claim would assume a very demanding notion of understanding according to which, for instance, an agent should offer a perfectly consistent analysis of numerous sacrificial dilemmas to understand the original utilitarian inference (in the Trolley dilemma). But this is unrealistic. Understanding comes in degrees and should not be entirely eliminated by occasional inconsistencies. So, inconsistencies typically reduce or limit, rather than undermine one’s original understanding.

Second, admittedly, one’s understanding is not *necessarily* reduced when opposite conclusions are inferred. On the one hand, “Pushing one person to save five is not morally acceptable” may improve understanding of the original utilitarian inference in the Trolley dilemma, if the agent can justify that there is, indeed, a moral difference between redirecting and pushing. On the other hand, even if the agent cannot identify a moral difference, inconsistency can perhaps demonstrate her understanding of the moral complexity of sacrificial dilemmas. However, this is simply not the type of understanding we are currently interested in. As stressed in Sect. 3, agents can understand issues, subject matters, concepts, events, etc., but our analysis focuses only on a specific type of understanding, i.e., “understanding why” some singular proposition is true. So, can inferring “We shouldn’t push one person off the bridge to save five” indicate or increase the agent’s understanding of “We should redirect the trolley and kill one person to save five due to the utilitarian maximization principle?” We can’t see how this is possible. Assuming that the agent is not able to explain how pushing changes the original utilitarian calculus associated with redirecting the trolley, unwarranted inconsistency will reduce, rather than increase her understanding why.

Third, contrary to the hypothesis presented above, the demandingness of sacrificial dilemmas cannot fully explain doubts and inconsistencies. Namely, if it could, moral agents would revise their judgments to a similar degree regardless of which moral dilemma was presented first.^17^ However, presenting the Trolly dilemma first leads to revisions more often (see Schwitzgebel and Cushman 2012).

This brings us to our fourth point. Even if inconsistency shows an understanding of sacrificial dilemmas, understanding is still affected by morally irrelevant variables. For instance, agents will understand less if first presented with the Footbridge dilemma because such an order of presentation leads to inconsistent judgments, doubts, and revisions less frequently. This only strengthens our original point because even if our definition is (partly) inadequate, and understanding can also be shown by doubts, hesitations, and inconsistencies, understanding remains (negatively) influenced by morally irrelevant variables. Modifying the definition does not avoid the problem. Accordingly, sensitivity to irrelevant variables poses a genuine challenge to understanding and accounts of moral progress based on such an ability. Our cognitive limitations—explainable in naturalistic terms—threaten understanding as much as knowledge.

It is also important to note that moral understanding naturally explains the expansion of the moral circle. While current scholarship recognizes moral inclusivity as one of the fundamental types of moral progress (see Buchanan and Powell 2018; Sauer et al. 2021), it overlooks the underlying cognitive mechanism that enables it. Our analysis of understanding fills this gap, contributing to the ongoing discussions about moral progress. Namely, understanding why, seen as the ability to adequately judge relevance (“Gender differences are morally irrelevant”) and reason consistently in new cases (“If men can vote, so can women”), is the epistemic precondition and causal explanation of moral inclusion. Furthermore, previous analysis shows that evolutionary constraints, flawed relevance assessments, and inconsistent reasoning threaten kinds of moral progress that do not require any moral inclusion. For instance, understanding sacrificial dilemmas does not involve moral reasoning with new *entities* (e.g., people of different races, genders, ethnicities, etc.), but in new *situations* (from “hitting the switch” to “pushing”). Accordingly, we demonstrate that progress in moral understanding requires not only expanding the moral circle of entities but also applying moral principles consistently across different situations.

In sum, understanding seems as threatened by the evolutionary origins of human cognition as knowledge: as long as understanding requires relevance-sensitive reasoning, UAP are not superior. But even if knowledge and understanding are equally challenged by natural selection, could modeling moral progress in terms of understanding still have some comparative benefits?

### UAP are (less) Demanding?

Another potential advantage of UAP concerns demandingness. According to the standard definition, to know why stealing is immoral, an agent must believe (instead of merely assuming) the proposition that is both true and morally justified (e.g., by private property rights). Also, justification should be neither defeated (e.g., by “Robin Hood” considerations) nor epistemically lucky. But all of this might be a little too much. Typical agents are not deeply reflective ethicists, but ordinary people mostly unaware of the subtle complexities of moral life. Can they regularly acquire something as demanding as knowledge? Are KAP, perhaps, fairly unrealistic?

UAP might be more adjusted to ordinary agents since understanding why *p* entails fewer requirements: true beliefs, sensitivity to defeaters, and absence of luck are not as strictly needed. In fact, if epistemic luck were a deal breaker, many clear cases of moral progress wouldn’t count as such. Suppose a racist father teaches his son “You must be kind to John because one should respect his neighbors.” The son morally improves although his progress is a matter of luck: namely, if, by chance, John were of a different race, the son would have learned a very different (immoral) lesson. Generally, much of our historical moral progress—like the reduction in violence, discrimination, etc.—was at least partially accidental, circumstantial or consisted of non-moral advances (Buchanan and Powell 2018). Although, ideally, moral progress should not be a matter of luck, it often does not include clean reasoning processes. Rather it is a messy combination of moral and non-moral factors that, in this case, improve people’s moral beliefs. So, if KAP cannot explain this “messiness”—e.g., how trusting unreliable sources often leads to moral improvement—perhaps UAP is a better alternative.

Nevertheless, on balance, the notion of understanding does not entail a more realistic account of moral progress. Arguably, understanding is more demanding than knowledge for at least three reasons. The first two concern the conditions for having moral understanding while the third reason concerns the ability to transfer it.

Understanding “*p* because *q*” requires grasping, i.e., the ability to arrive at correct judgments when variables change. But slightly changed variables often lead to moral dilemmas. Consider Plato’s famous example. “Returning a borrowed weapon to a friend (let’s name him Mark) is just (*p*) because of one’s obligation to repay debts (*q*)” is an uncontroversial inference that, nevertheless, might easily become a moral dilemma: we only need to imagine that Mark is mentally ill, i.e., change “Returning a borrowed weapon to a friend” with a slightly different variable “Returning a borrowed weapon to a *mentally ill* friend.” Furthermore, we can also imagine that Mark stole the weapon from John. So, are we obliged to return it? And to whom: Mark, or John? To make arriving at correct moral judgments difficult, we only need to *imagine* some changes in morally relevant variables, and, sometimes, even manipulating morally irrelevant variables might create challenges (recall the Trolley vs. Footbridge dilemma).^18^

If great ethical minds cannot agree on how certain dilemmas should be resolved (see McConnell 2022), it is extremely unrealistic to expect ordinary agents to handle them competently.^19^ Is, then, better to model moral progress in terms of knowledge, after all? We formulate two reasons why knowing is less challenging than understanding.

First, knowledge can be “easy.” Often, knowing does not entail resolving moral dilemmas since, in normal circumstances, our friends are not mentally ill, or thieves. So, knowing why we should return borrowed items comes easily: if my friend is in the right mind, I know why returning the weapon is right, even if I cannot deal with a tricky hypothetical case of mental illness. By contrast, understanding cannot be easy in a comparable sense because grasping requires the ability to reason in hypothetical cases and variables that do not arise in actual circumstances can simply be imagined. So, even if, de facto, my friend Mark is in the right mind, I must be able to deal with a hypothetical case where Mark is mentally ill to understand that “Returning a borrowed weapon to Mark is just because…” Since the actual absence of tricky variables does not affect grasping, there are always hypothetical cases to consider and understanding cannot be easy.

Second, even when they concern dilemmas, knowledge conditions are relatively straightforward. When our inference “Returning a borrowed weapon to Mark is just because…” gets challenged by “Mark is mentally ill,” we know that, essentially, the right judgment comes down to choosing between two norms: repaying debts and avoiding harm. As soon as we know which norm is preferable, we’ll know whether returning the weapon is morally right. By contrast, it is unclear how many different value conflicts and hypothetical dilemmas an agent should manage to understand the original inference. Suppose I reason correctly when Mark is mentally ill, but underperform when he is a thief. Do I understand the original inference? Or suppose I draw correct conclusions in cases of mental illness and theft, but misjudge the situation when the weapon is much more useful to me (e.g., when that is my only weapon during the war, while Mark also owns another one). Without specifying “how much” grasping counts as understanding, understanding is difficult to ascribe, and easy to deny. So, UAP might struggle with determining whether moral progress has occurred or not.^20^

All of this shows that, compared to knowledge, understanding requires broader comprehension of a subject matter (Elgin 2007; Grimm 2021) and some creative imagination (Moody-Adams 2017) that “enables non-trivial inference” (Elgin 2007, p. 39). If understanding just one inference requires accurate answers to many hypothetical questions (Hills 2016), then the ultimate object of understanding is more complex than the one of propositional knowledge (Elgin 2007; Zagzebski 2019). If this is so, then, usually, understanding is more difficult to acquire than knowledge; represents a greater cognitive achievement (Pritchard 2010); and, as a result, leads to more demanding accounts of moral progress.

The third reason why understanding is more demanding concerns transmissibility by testimony and this is relevant since transmissibility of moral information and skills are recognized as important mechanisms of moral progress (think, for example, about moral education). Social epistemologists mostly agree that testimony can transmit (Hardwig 1985; Audi 1997) or generate defeasible knowledge (Lackey 2008): if Theresa is a trustworthy moral expert who testifies “Stealing is immoral because it violates the right to private property,” and John—who is not gullible—defers to her opinion, then, presumably, John knows why stealing is immoral. Testimony transmits moral knowledge (see Hills 2009; McGrath 2019).

But if Theresa is also able to grasp why stealing is immoral, her testimony does not transfer her intellectual ability to John. John can defer to her opinion while remaining completely incompetent to judge hypothetical cases like Mark not returning the money, or the corporation’s usurpation of public property. On the one hand, a single testimony cannot transfer a broader comprehension of a subject matter and, on the other, communication cannot directly transfer abilities. To be sure, experts can explain how things are done, but practical skills—e.g., cooking, driving a car, or reasoning well in new circumstances—are developed through training, experience, and personal effort (Hills 2009, 2016). So, abilities get lost in transmission: while testimony may induce, facilitate, or speed up someone’s understanding, it cannot, strictly speaking, transfer it. Understanding is more individualistic than knowledge: grasping is something we can only do “first hand” (Zagzebski 2008).^21^

This has direct repercussions on the account of moral progress since ordinary agents are social beings, and theorists may seek to develop an account that, in principle, can also be applicable at the societal level. However, societies can hardly progress morally without efficient social learning processes such as moral education, and the latter mostly depends on trusting expert testimonies. The transmission of epistemic goods—such as truth, knowledge and understanding—is the traditional goal of education (Watson 2016, p. 152) and the increase in these types of goods can be considered progress. Some scholars believe that the main educational goal should be understanding because “something over and above the mere transmission of truth or knowledge is required in order to satisfy the proper aims of teaching and learning” (Watson 2016, p. 153), but we should keep in mind that, for reasons mentioned above, understanding is much more difficult, if not impossible to transfer.^22^ Also, exceptional circumstances might require an urgent moral response (think about fighting climate change). In such cases, expert testimony can facilitate moral progress by quickly transmitting actionable knowledge, even if it cannot transmit the first-hand intellectual ability to deal with numerous hypothetical cases.

Admittedly, it is hard to say if, on balance, understanding or knowledge is more demanding since demandingness comes in different forms that cannot be easily compared. While knowing why entails several conceptual conditions, understanding why (always) requires the ability to handle difficult cases, and seems theoretically open-ended: even when an agent faces an actual dilemma, it is more difficult to pinpoint what, exactly, should she do to understand, than what should she do to know. So, while knowing is conceptually complicated but, at least sometimes, cognitively easy, understanding is, arguably, conceptually simpler but (almost) always cognitively hard. Moreover, an ability to understand cannot be directly transferred via testimony, a ubiquitous epistemic source. This makes it difficult to acquire and spread, thereby posing an empirical challenge to social theories of moral progress. Saying that UAP are at least as demanding as KAP seems like a fair verdict at this point.

### UAP Capture the Irreversibility of Moral Progress?

The last potential advantage of UAP discussed here concerns cognitive stability of understanding. Namely, once developed, abilities tend to stick around.^23^ If you can type by using just two fingers, learning new information or skill will not eliminate this ability. Similarly, once you understand some theory, you won’t immediately lose the ability to manage its variables after acquiring new information, or by understanding some conflicting theory. Understanding is a relatively stable epistemic achievement. By contrast, knowledge seems fragile.^24^ If you know “*p* because *q*” at *t*_*1*_, the proposition’s epistemic status might easily change at *t*_*2*_ due to acquiring a defeater, irresponsible treatment of evidence, or learning about epistemic luck.^25^

So, while, compared to knowledge, understanding is more demanding to acquire and transfer, it seems easier to preserve. Cognitive resistance of understanding—recognized by authors like Lipton (2009), Hills (2016), and Severini (2021)—is important for modeling moral progress since several authors suggest that genuine progress should be irreversible. That is, progress is usually described as durable, non-superficial, and irreversible change, in the sense that it cannot be undone without difficulty (Schinkel and de Ruyter 2017).

Someone who, through serious practice, makes progress at the piano, may fall back through lack of practice, but it is unlikely that she will return to her pre-practice level, unless she does not play the piano for a substantial period of time; furthermore, the fact that she will most likely need less practice than the first time to regain her previous top level, suggests that an ‘internal’ change has indeed taken place. (Schinkel and de Ruyter 2017, p. 123)

According to a strict understanding of moral progress, not every change for the better counts as true or genuine moral progress (Buchanan and Powell 2018). Superficial and easily reversible changes will not make the cut. So, if understanding, rather than knowing, allows us to keep what we already have, are UAP superior to KAP? On balance, they are not.

While understanding’s cognitive resistance avoids cases of undesirable moral change (regressing to the previous state), we believe it cannot naturally explain many paradigmatic cases of doxastic change, i.e., cases of moral progress where prior beliefs get abandoned or revised. Once we used to find slavery acceptable, but today we reject it. Once we used to believe that only men should participate in public life, but today we recognize equal rights for women, etc. While revising moral beliefs is difficult (see Horne et al. 2015), KAP at least recognize its relevance and show how new information (e.g., defeaters) should change our previous beliefs. By contrast, UAP struggle to explain belief revisions for two interconnected reasons.

First, the very definition of grasping reveals that belief revision is inessential for understanding why. Grasping “*p* because *q*” requires dealing with similar principles (“p* because q*”) or applying the original principle to new circumstances, but does not explicitly mention any abilities focused on criticizing, testing, revising, or abandoning “*p* because *q*.” Second, since understanding is sometimes insensitive to evidence, it cannot consistently explain the cases of moral progress that, in fact, result from considering new evidence. For instance, imagine that our moral belief “Killing animals is acceptable because they do not have the capacity to suffer” is challenged by the new evidence _*C*_: “Scientific research proves that animals can suffer.” We might morally progress by rejecting the original conclusion (“Killing animals is acceptable”) in light of *c*, or, perhaps, keeping the original inference by defeating _*C*_ (e.g., showing that scientific research was, in fact, methodologically flawed). However, in both cases the progress results from considering the new evidence. As explained in Sect. 4.3, this requirement is challenging for UAP because understanding, unlike knowledge, can be insensitive to defeaters and epistemic luck.

However, we do not suggest that understanding is completely insensitive to evidence and entirely independent of epistemic justification. According to moderate non-factive accounts, understanding is positively related to truth (Elgin 2007), and truth is closely connected to justification. Also, there is no reason to think that grasping “*p* because *q*” only requires the ability to reason correctly with *revised* variables (p* and q*), but not with *additional* variables (e.g., with *p*, *q*, and a defeater *c*). However, the exact relevance of epistemic justification is difficult to specify since justifying remains unnecessary for understanding. As shown in Sect. 4.3, understanding can rely on approximations and idealizations (Elgin 2007), and be insensitive to misleading evidence and epistemic luck (Hills 2016). The relationship between justifying and knowing is clearer because, on standard view, justification (of some kind, e.g., internalist, or externalist) is necessary for knowledge.

So, although we do not claim that UAP can *never* adequately explain the doxastic change, we are under the impression that explaining the doxastic change systematically and consistently is more difficult to UAP than KAP. If the understanding is an ability that does not automatically change with new information, how can UAP explain belief revision? And if understanding does not necessarily require evidential justification, how can UAP consistently and reliably explain doxastic change resulting from an evidential update? These questions might have good answers, but until such answers are presented, KAP appear more suitable for explaining moral progress via belief revision.

To conclude, the stability of understanding coheres well with the intuition that some moral achievements are permanent or non-retractable. However, due to cognitive resistance, understanding cannot elegantly explain progressing via previous belief revision. Since belief revision is quite an elementary mechanism of epistemic moral progress, KAP are indispensable in developing a plausible theory of moral progress, and, arguably, more promising for capturing its internal dynamics.

## Concluding Remarks

The literature on moral progress contends that a plausible theory of moral progress must “identify at least the most important types of moral progress; […] determine whether there are interesting conceptual, normative, or causal relations among them; and […] provide significant practical guidance as to how these types of moral progress can be achieved” (Buchanan and Powell 2018, p. 31). This paper advances current scholarship by distinguishing between understanding- and knowledge-based theories of moral progress, and by examining their features and interrelationships in more detail and from new perspectives.

Knowledge and understanding are importantly different, but both represent desirable cognitive outcomes of moral reasoning. Unlike Severini (2021), we believe that, in principle, both goals are attainable by ordinary agents. However, the questions that remain to be settled are: under what conditions, how easily, and at what costs can ordinary moral agents acquire moral knowledge and understanding? These questions reveal a more practical, non-ideal route we took in our previous analysis. We focused on comparing different epistemic (and, consequently, moral/practical) risks associated with knowledge and understanding, rather than abstract metaethical issues. We argued that both UAP and KAP show potential in explaining how individual moral reasoning can be improved. However, in response to Severini’s recent argument about UAP’s superiority, we showed that not only UAP face similar challenges as KAP, but they also face some distinctive, additional difficulties.

Due to the epistemic unreliability of belief-forming processes, the evolutionary explanations of morality pose serious skeptical challenges to KAP. However, there is no a priori reason to believe that UAP are immune to various epistemic limitations of human cognition, also explainable in evolutionary terms. In fact, scientific studies on solving sacrificial dilemmas suggest that our ability to have moral understanding is at least equally affected by the evolutionary origins of morality. Furthermore, UAP face some additional difficulties due to the comparative demandingness of understanding. For instance, understanding, unlike knowledge, (always) requires competent reasoning about hypothetical moral dilemmas. Also, unlike knowledge, understanding is not directly transmissible by testimony, which hinders theories of moral progress focused on social learning processes. Knowledge appears to be more accessible than understanding which is an important advantage in combating pressing moral challenges of the modern world. Finally, due to the cognitive resistance of understanding, UAP struggle to explain belief revisions. Therefore, theories focusing on promoting moral progress may find KAP more convincing.

Overall, we believe that, at this stage, KAP show more potential than UAP. They are not more threatened by the evolutionary origins of (moral) reasoning than UAP, but provide clearer theoretical criteria for progress, are more capable of explaining doxastic change, and can be generalized more naturally on a societal level. Since understanding is a more private, “first person” ability, even if societies may know, they can hardly understand. KAP also seems less demanding, and even when they are more demanding, this is for a good reason. Arguably, a moral agent’s sensitivity to truths and defeaters, and her epistemic responsibility are typically more important than her ability to deal with tricky hypothetical cases.

While our focus was on moral progress, our analysis of cognitive limitations, consistency preservation, doxastic change, reasoning in new circumstances, epistemic transferability, etc., is relevant to anyone interested in moral reasoning. It is relevant to scholars interested in abstract metaethical debates concerning moral ontology (realism vs. anti-realism) and epistemology (cognitivism vs. non-cognitivism), as well as to those interested in more practical debates (concerning, e.g., moral enhancement, or moral education).

## Data Availability

Not applicable.

## Footnotes

1 Irreversible in the sense that it cannot be wholly undone without difficulty. This especially applies to considered judgments of progress where change is an “external expression of an internal or underlying change” (Schinkel and de Ruyter 2017, p. 123).

2 Not everyone agrees that moral reasoning plays an important role in moral progress or that it can explain major episodes of progressive moral change. For such views see, e.g., Rehren and Blunden (2024).

3 We are not concerned here with the question of how much moral progress can be explained, or how much moral progress is caused by moral reasoning. We do believe that at least some amount of moral progress comes about through moral reasoning but we won’t be taking a stance on whether this is sufficient to cause group-level or large-scale moral improvement. Instead, we focus on how individual-level changes in moral reasoning contribute to moral progress and whether they are, epistemically speaking, better explained in terms of knowledge or understanding.

4 Presumably, moral epistemologists can rely on definitions proposed by general epistemologists since “the standards for moral knowledge … are not materially different from the standards for knowledge in general” (McGrath 2019, p. 6).

5 This paper focuses on the so-called propositional (theoretical, cognitive) knowledge, i.e., an epistemic state that ties our beliefs to the state of affairs by correspondence or representation (broadly understood). It does not focus on practical knowledge (“know how”), i.e., abilities required to perform an action that changes the state of affairs.

6 Obviously, defeater-sensitivity applies only to defeasible knowledge. The question of whether all (moral) beliefs are defeasible (as fallibilist accounts suggest), or are there also irrebuttable, conclusively-justified beliefs, epistemic starting points, (moral) axioms, etc., falls outside of the scope of this paper.

7 Similarly, reliable perception can justify the belief “It is raining outside,” but does not generate knowledge *why* it is raining outside. To have this sort of knowledge, the subject must refer to other causally-relevant natural phenomena, such as the water cycle, clouds and water droplets, condensation, gravity, etc.

8 To show understanding, John can paraphrase or expand the original inference, e.g.: “The private property represents a human right, and a cornerstone of liberal democracies. Thus, whatever threatens this right—ignorance, aggression, violence, or, perhaps, stealing—must be seen as morally problematic.”

9 Not only manipulation, but applying to different contexts. To grasp “*p* because *q*,” one should be able to apply it in different areas and analyze new problems (Elgin 2007).

10 Leite (2004) argues, traditional accounts usually portray epistemic subjects as spectators, and knowledge as something that passively happens to them.

11 Potentially, the non-factive notion of understanding might benefit the theory of moral progress in two additional ways. First, according to Severini (2021), non-factive understanding avoids a commitment to moral realism, a fairly controversial metaethical theory. However, this view is problematic since moderate non-factive accounts explicitly acknowledge the existence of truths and facts. Although understanding is not “slavish … to the facts” (Elgin 2007, p. 33), it usually relies on idealizations that produce truths, or propositions that are approximately true. Second, in our opinion, setting moral truths aside might be necessary to explain theoretical progress. Elgin (2007) argues that scientific progress cannot be explained in terms of increased knowledge because, strictly speaking, scientists move from one false theory to another. In line with the so-called pessimistic induction, all previous scientific theories were false, so our current theories are probably false, too. Can this argument be applied to ethics as an academic discipline? If ethical progress involves changing false moral theory *T*_*1*_ for false (but more plausible) theory *T*_*2*_, then explaining progress in terms of the non-factive notion of understanding appears more promising.

12 As Quine famously remarked, “[c]reatures inveterately wrong in their inductions have a pathetic but praiseworthy tendency to die before reproducing their kind” (1969, p. 126). In a narrower discussion concerning moral knowledge, Wielenberg (2010), Brosnan (2011), and Shafer-Landau (2012) offer several arguments showing consistency between knowledge and natural selection.

13 For our discussion, it is important to notice that these biases seem damaging to understanding, as well as knowledge.

14 For instance, if, while reasoning about the new case, we change our original conclusion that “Stealing from John is immoral” due to the morally irrelevant variable “John’s middle name is Anthony,” then our understanding of the original inference is compromised.

15 As Kumar and May explain, some studies (e.g., Mikhail 2014) suggest that judgments about sacrificial dilemmas are not simply emotional aversions to up-close and personal harm but that participants seem to be unconsciously applying rules, such as a prohibition on battery, prototypical violence, using another person as a means to one’s ends, or some combination of these (for more, see Kumar and May, Forthcoming).

16 We thank the anonymous reviewer for pushing us to clarify this point.

17 Plausibly, the order of presentation cannot determine the objective moral demandingness of the Trolley, or the Footbridge dilemma.

18 Importantly, understanding the connection “*p* because *q*” requires that variables are changed in a relevant, meaningful way which introduces new moral challenges. If circumstances are only trivially changed—e.g., a friend’s name is Steve (instead of Mark), or Mark is a neighbor (instead of a friend)—an agent will not demonstrate her deeper moral understanding of “Returning a borrowed weapon to a friend Mark is just because of one’s obligation to repay debts” by reasoning correctly with changed variables. So, moral understanding might not only include, but also *require* the ability to deal with moral dilemmas.

19 Also, studies have shown that even philosophy experts can be biased, manipulated or swayed by irrelevant factors and their reasoning is unreliable in solving moral dilemmas (see, e.g., Schwitzgebel and Cushman 2012; 2015).

20 Admittedly, this is more of a theoretical, than a practical problem. Understanding can increase when one becomes more competent to discuss some hypothetical case, even if she cannot *resolve* it. Also, understanding comes in degrees, and, sometimes, is obvious: if somebody can handle ten moral dilemmas, she surely understands the original inference to a remarkable degree, even if she cannot resolve the eleventh. However, defining what should count as understanding is still necessary if UAP attempt to provide a precise, elaborate, theoretically solid theory of moral progress.

21 Differences between understanding and knowledge might be relevant for studying moral motivation, another essential aspect of moral progress recognized in the literature (see Buchanan and Powell 2018, p. 55; Sauer et al. 2021, p. 4). Since antiquity, it has been recognized that knowledge alone does not reliably lead to moral action, as highlighted in discussions of “weakness of will” (*akrasia*). Our guess is that understanding might be a more desirable cognitive achievement as far as moral motivation is concerned. Since it assumes a deeper comprehension of morality, requires more time and effort, and is somehow more “personalized” than knowledge, presumably, understanding why something is morally right may increase one’s *intrinsic* motivation to act accordingly. However, this is only a commonsensical hypothesis that warrants further empirical testing.

22 That understanding is rather individualistic and not easily transferred from person to person may be a reason to think that some kinds of moral progress should be studied individualistically, rather than socially. This goes against those who believe that moral progress is inherently social (see Sauer et al. 2021, p. 3).

23 Hence the phrase “It’s like riding a bike!”

24 As de Regt (2015, p. 3782) remarks, after learning that Einstein’s theory of general relativity refutes the existence of attractive forces, a physicist is still capable to use Newton’s theory of gravitation. The latter theory does not represent knowledge, but, as an approximation, remains helpful in understanding the gravitational phenomena.

25 Some theorists believe that moral knowledge is, in fact, less fragile than other kinds of knowledge (e.g., empirical knowledge). Together with McGrath (2019, p. 3), we believe that this isn’t the case. But this is not a central question for our purposes, anyway. The central question is: is moral knowledge less fragile than moral understanding? We believe it is (for reasons mentioned in Sect. 4.3).
